# Paracrine- and cell-contact-mediated immunomodulatory effects of human periodontal ligament-derived mesenchymal stromal cells on CD4^+^ T lymphocytes

**DOI:** 10.1186/s13287-024-03759-4

**Published:** 2024-05-31

**Authors:** Christian Behm, Oliwia Miłek, Xiaohui Rausch-Fan, Andreas Moritz, Oleh Andrukhov

**Affiliations:** 1 Competence Center for Periodontal Research, University Clinic of Dentistry, Medical University of Vienna, Sensengasse 2A, 1090 Vienna, Austria; 2 Clinical Division of Conservative Dentistry and Periodontology, University Clinic of Dentistry, Medical University of Vienna, Sensengasse 2A, 1090 Vienna, Austria; 3 Center for Clinical Research, University Clinic of Dentistry, Medical University of Vienna, Sensengasse 2A, 1090 Vienna, Austria

**Keywords:** Human periodontal ligament-derived mesenchymal stromal cells, TNF-α, IL-1β, Immunomodulation, CD4^+^ T lymphocytes, Co-culture

## Abstract

**Background:**

Mesenchymal stromal cells (MSCs) isolated from the periodontal ligament (hPDL-MSCs) have a high therapeutic potential, presumably due to their immunomodulatory properties. The interaction between hPDL-MSCs and immune cells is reciprocal and executed by diverse cytokine-triggered paracrine and direct cell-to-cell contact mechanisms. For the first time, this study aimed to directly compare the contribution of various mechanisms on this reciprocal interaction using different in vitro co-culture models at different inflammatory milieus.

**Methods:**

Three co-culture models were used: indirect with 0.4 μm-pored insert, and direct with or without insert. After five days of co-culturing mitogen-activated CD4^+^ T lymphocytes with untreated, interleukin (IL)-1β, or tumor necrosis factor (TNF)-α- treated hPDL-MSCs, the CD4^+^ T lymphocyte proliferation, viability, and cytokine secretion were investigated. The gene expression of soluble and membrane-bound immunomediators was investigated in the co-cultured hPDL-MSCs.

**Results:**

Untreated hPDL-MSCs decreased the CD4^+^ T lymphocyte proliferation and viability more effectively in the direct co-culture models. The direct co-culture model without inserts showed a strikingly higher CD4^+^ T lymphocyte cell death rate. Adding IL-1β to the co-culture models resulted in substantial CD4^+^ T lymphocyte response alterations, whereas adding TNF resulted in only moderate effects. The most changes in CD4^+^ T lymphocyte parameters upon the addition of IL-1β or TNF-α in a direct co-culture model without insert were qualitatively different from those observed in two other models. Additionally, the co-culture models caused variability in the immunomediator gene expression in untreated and cytokine-triggered hPDL-MSCs.

**Conclusion:**

These results suggest that both paracrine and cell-to-cell contact mechanisms contribute to the reciprocal interaction between hPDL-MSCs and CD4^+^ T lymphocytes. The inflammatory environment affects each of these mechanisms, which depends on the type of cytokines used for the activation of MSCs’ immunomodulatory activities. This fact should be considered by comparing the outcomes of the different models.

**Supplementary Information:**

The online version contains supplementary material available at 10.1186/s13287-024-03759-4.

## Background

Mesenchymal stromal cells (MSCs) reside in the perivascular area [1, 2] of various tissues throughout the human body, including the bone marrow, umbilical cord, and adipose tissue [3, 4]. Additionally, they can also be isolated from various dental tissues, such as the periodontal ligament (PDL) [5], which functions as a connective tissue between the jawbone and cementum [6]. Like MSCs from the bone marrow, human periodontal ligament-derived mesenchymal stromal cells (hPDL-MSCs) comply with the minimal criteria for MSCs [3], showing a plastic adherence and tri-lineage differentiation potential, and the expression and lack of mesenchymal and hematopoietic surface markers, respectively [7]. Many studies discuss the transplantation of MSCs isolated from various tissues, including the PDL, as a treatment option against various degenerative and inflammatory diseases [8–10]. This therapeutic potential is primarily based on the hPDL-MSCs’ capability to modulate the activity of various cells of the innate and adaptive immune system [11].

Two different groups of mechanisms are largely responsible for these immunomodulatory abilities: (1) the secretion of soluble immunomediators and enzymes, including indoleamine-2,3-dioxygenase-1 (IDO-1), prostaglandin E_2_ (PGE_2_), and tumor necrosis factor-inducible gene 6 protein (TSG-6), which regulate immune cells in a paracrine fashion; (2) and direct cell-to-cell contact mechanisms by expressing membrane-bound immunomediators, including programmed cell death ligand 1 (PD-L1) and PD-L2, coded by the genes *CD274* and *CD273*, respectively [3, 12]. These immunomodulatory mechanisms are usually low under homeostatic conditions but are boosted by an inflammatory environment via various pro-inflammatory cytokines, including interleukin (IL)-1β and tumor necrosis factor (TNF)-α [13, 14]. Since immune cells are the primary source of these pro-inflammatory cytokines, this leads to a tight bi-directional interaction between hPDL-MSCs and immune cells [15].

**Fig. 1:**
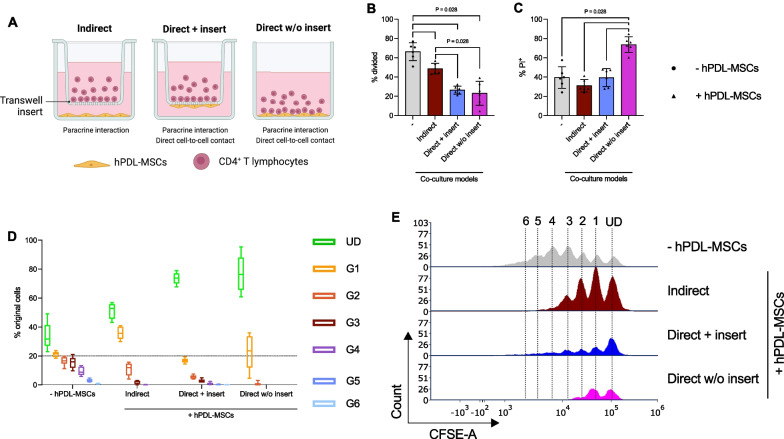
The ability of hPDL-MSCs to suppress CD4^+^ T lymphocytes varies depending on the co-culture model. Depending on the co-culture model, hPDL-MSCs were seeded in 6-well plates (indirect and direct w/o insert) or on the underside (direct + insert) of the TC insert’s membrane (**A**). PHA-L-activated CD4^+^ T lymphocytes were added to the TC inserts (indirect and direct + insert) or directly (direct w/o inserts) to the hPDL-MSCs. After five days of incubation, CD4^+^ T lymphocyte proliferation and viability were determined by CFSE and PI staining, respectively, followed by flow cytometry analysis (**B**–**E**). **B** reveals the percentage of original CD4^+^ T lymphocytes that have divided, whereas **C** shows the percentage of non-viable CD4^+^ T lymphocytes. The number of original CD4^+^ T lymphocytes per generation is presented as a percent of the total number of original CD4^+^ T lymphocytes for the undivided (UD) and each divided (G1–G6) generation (**D**). The numbers of CD4^+^ T lymphocytes in each generation (UD, and G1–G6) are shown in representative histograms (**E**). The data were obtained from six independent repetitions using hPDL-MSCs from a different individual per repetition. In **B**–**C**, data are presented as mean ± standard error of the mean (S.E.M) and as individual data points. In **D**, data are presented as box-whisker plots showing the median, minimum and maximum values. The statistical significance was evaluated by the Friedman Test, followed by the Wilcoxon Test for pairwise comparison. *P* values < 0.05 were statistically significant and were indicated between the groups (**B**–**C**)

Most of the knowledge about this reciprocal interrelation has derived from in vitro studies primarily using two different models to co-culture hPDL-MSCs with immune cells (Fig. 1A) [14, 16–19]. In the direct co-culture model, immune cells are added directly to plastic-adherent hPDL-MSCs (direct without insert) [17–19]. Another possibility for the direct co-culture model is the attachment of hPDL-MSCs to the bottom side of a porous membrane and the addition of immune cells to the opposite side directly into the insert, allowing limited cell interaction through membrane pores [20]. For the indirect co-culture model, hPDL-MSCs and immune cells are spatially separated by a liquid-permeable membrane (indirect with insert) [14, 16, 18]. All mentioned co-culture types are sophisticated and convenient models to explore the reciprocal interaction between hPDL-MSCs and immune cells and allow to discriminate between the different immunomodulatory mechanisms (Fig. 1A). The indirect co-culture model only allows a paracrine interaction between hPDL-MSCS and immune cells, hardly mimicking the in vivo situation, whereas the direct co-culture types additionally enable direct cell-to-cell contacts with a different degree depending on the settings [4]. Hence, combining the various co-culture models creates a better and more differentiated view of the in vivo situation and allows to estimate the contribution of the individual immunomodulatory mechanisms to the immunoregulating effects of hPDL-MSCs.

One of the most investigated immunoregulatory effects of MSCs is their interaction with T lymphocytes [3]. Numerous studies have already displayed the suppressive effects of hPDL-MSCs on the CD4^+^ T lymphocyte proliferation and the ability to trigger and inhibit regulatory CD4^+^ T lymphocyte (T_regs_) and CD4^+^ Th17 lymphocyte differentiation [14, 16–19]. However, these studies mainly used a single co-culture model [14, 16, 17, 19], incapable of discriminating between the paracrine and direct cell-to-cell contact immunomodulatory mechanisms of hPDL-MSCs. To the best of our knowledge, only one study displayed the immunosuppressive effects of hPDL-MSCs via the two co-culture models, however, using peripheral blood mononuclear cells (PBMCs) [18]. Hence, no study exists that directly compares the different co-culture models in the context of the reciprocal interaction between hPDL-MSCs and CD4^+^ T lymphocytes.

Hence, the main objective of this in vitro study was to compare the bi-directional interaction between hPDL-MSCs and CD4^+^ T lymphocytes using the direct and indirect co-culture models in different cytokine milieus. In detail, we explored CD4^+^ T lymphocyte proliferation, viability, and the secretion of CD4^+^ T lymphocyte subset-specific cytokines after co-culturing CD4^+^ T lymphocytes with unstimulated, IL-1β, or TNF-α-stimulated hPDL-MSCs in the three different co-culture models. Additionally, we directly compared the influence of the various co-culture models on the immunomodulatory mechanisms of hPDL-MSCs by investigating the immunomediator gene expression in hPDL-MSCs after the co-culture with CD4^+^ T lymphocytes. Our data reveal that the co-culture models differently affect the reciprocal interaction between hPDL-MSCs and CD4^+^ T lymphocytes, suggesting that both the paracrine and direct cell-to-cell immunomodulatory mechanisms contribute to the observed immunomodulatory activities of hPDL-MSCs against CD4^+^ T lymphocytes. This contribution seems to originate from the variability of the immunomediator expression in hPDL-MSCs depending on the co-culture model and the present cytokine type.

## Methods

### *Ethical statement*

The Ethics Committee of the Medical University of Vienna approved the isolation of MSCs from the PDL and CD4^+^ T lymphocytes from whole blood (EK Nr.: 1694/ 2015, valid up to 10/2024). The isolation and experimental protocols were conducted in accordance with the Declaration of Helsinki and the Good Scientific Practice Guidelines of the Medical University of Vienna.

### *hPDL-MSCs isolation and cultivation*

Primary MSCs were isolated from the PDL of third molars donated from six different periodontally healthy individuals. These patients underwent tooth extraction for orthodontic reasons and gave written informed consent before the surgical procedure. The PDL was scrapped off the mid-third of the tooth’s roots, chopped, and cultivated in Dulbecco’s Modified Eagle’s medium (DMEM, Capricorn Scientific GmbH, Ebsdorfergrund, Germany) supplemented with 4.5 g/ml glucose, L-glutamine, 10% fetal bovine serum (FBS, Gibco, Carlsbad, USA), 50 μg/ml streptomycin (Gibco), and 100 U/ml penicillin (Gibco). The tissue pieces were incubated at 37 °C, 5% carbon dioxide, and 95% humidity until hPDL-MSCs had grown out of the PDL tissue. Isolated hPDL-MSCs were sub-cultured under the same culture conditions and used for experiments between passages five and seven.

According to the International Society for Cell and Gene Therapy [7], isolated hPDL-MSCs correspond with the minimal criteria for MSCs, expressing specific surface markers (CD29, CD90, CD105, and CD146) and lacking the expression of hematopoietic markers (CD14, CD31, CD34, and CD45) as described in our previous studies [14, 21].

### *Allogeneic CD4*^+^*T lymphocyte isolation*

Whole blood was collected using a heparin- and lithium-containing VACUETTE^®^ blood collection system (Greiner Bio-one, Kremsmünster, Austria), puncturing the median cubital or cephalic vein from one volunteer throughout the study. The heparinized blood was diluted 1:1 with Hank’s Balanced Salt Solution (HBSS, Life Technologies, Carlsbad, CA, USA), followed by density gradient centrifugation using Ficoll-Paque (GE Healthcare, Chicago, IL, USA). After washing PBMCs with HBSS and resuspending in 1xphosphate buffered saline (1xPBS), CD4^+^ T lymphocytes were isolated by the negative immunomagnetic selection method using the MagniSort™ Human CD4^+^ T cell enrichment kit (Invitrogen, Carlsbad, CA, USA). Isolated CD4^+^ T lymphocytes were cultured at 37 °C, 5% carbon dioxide, and 95% humidity using RPMI-1640 medium (Sigma-Aldrich, St. Louis, MO, USA), supplemented with L-glutamine, sodium bicarbonate, 10% FBS, 50 μg/ml streptomycin, and 100 U/ml penicillin. The purity of enriched CD4^+^ T lymphocyte populations (93%) was verified in our previous study [22] by staining the CD4 antigen followed by flow cytometry analysis.

### *Co-culture models and cell treatments*

Primary hPDL-MSCs and allogeneic CD4^+^ T lymphocytes were co-cultured (1:4) using three different co-culture models (Fig. 1A):

1. Indirect co-culture: 2.5 × 10^5^ hPDL-MSCs were added per well in 6-well plates using fully supplemented DMEM and were incubated for 24 h before pre-stimulation with 5 ng/ml IL-1β, or 10 ng/ml TNF-α (both from Peprotech, London, Great Britain) using complete DMEM without FBS. hPDL-MSCs without any cytokines served as control. After 48 h of incubation, hPDL-MSCs were re-stimulated as described above. Freshly isolated, allogenic 1 × 10^6^ CD4^+^ T lymphocytes were added into transwell (TC) inserts with a pore size of 0.4µm (Sarstedt, Biedermannsdorf, Austria). hPDL-MSCs without CD4^+^ T lymphocytes were a control for the immunomediator gene expression analysis in hPDL-MSCs. Fully supplemented RPMI-1640 was used for hPDL-MSCs’ re-stimulation and CD4^+^ T lymphocyte seeding. CD4^+^ T lymphocytes were activated by 10 μg/ml phytohemagglutinin-L (PHA-L, Thermo Fisher Scientific, Waltham, MA, USA). PHA-L-activated CD4^+^ T lymphocytes in the absence of hPDL-MSCs served as control.
2. Direct co-culture with inserts: The membrane underside of the TC inserts was used to seed 1 × 10^5^ hPDL-MSCs using fully supplemented DMEM followed by an incubation time of 24 h and a stimulation as described above. After 48 h of incubation, hPDL-MSCs were re-stimulated as described above, and 4 × 10^5^ PHA-L- (10 µg/ml) activated CD4^+^ T lymphocytes were added into the TC inserts. The same controls, as for the indirect co-culture model, were used.
3. Direct co-culture without inserts: 2.5 × 10^5^ hPDL-MSCs were seeded per well in 6-well plates using fully supplemented DMEM. After 24 h of incubation and a 48 h pre-stimulation period, hPDL-MSCs were re-stimulated with cytokines as described above. Per well, 1 × 10^6^ PHA-L- (10 µg/ml) activated CD4^+^ T lymphocytes were added directly to the hPDL-MSCs. The same controls, as for the other models, were used.

After 5 days of incubation in the three different co-culture models, the proliferation of viable CD4^+^ T lymphocytes, the percentage of non-viable CD4^+^ T lymphocytes, and the secretion of CD4^+^ T lymphocyte-specific cytokines were determined by carboxyfluorescein succinimidyl ester (CFSE), and propidium iodide (PI, both from Thermo Fisher Scientific, Waltham, MA, USA) staining, and cytokine multiplexing (LEGENDplex™, BioLegend, San Diego, CA, USA), respectively, using the flow cytometry analysis. Immunomediator gene expression in hPDL-MSCs was determined by quantitative polymerase chain reaction (qPCR).

### *CD4*^+^*T lymphocyte proliferation and viability analysis*

Freshly isolated, allogenic CD4^+^ T lymphocytes were stained with the CellTrace™ CFSE Cell Proliferation kit (Thermo Fisher Scientific, Waltham, MA, USA) according to the manufacturer’s instructions. In brief, CD4^+^ T lymphocytes were resuspended with 1xPBS containing 5% FBS, reaching a final cell concentration of 1 × 10^6^ cells/ml. CD4^+^ T lymphocyte suspension was stained with 2.5 μM CFSE for 5 min at room temperature. After a washing step with fully supplemented RPMI-1640 and a resting period of 10 min at 37 °C, CFSE-labelled CD4^+^ T lymphocytes were added to the hPDL-MSCs using the three different co-culture models.

After five days of incubation, CD4^+^ T lymphocytes were harvested, washed with 3% bovine serum albumin (BSA, Merck Millipore, Burlington, USA; + 0.09% sodium azide in 1xPBS), and labelled with 20 μg/ml propidium iodide (PI, Thermo Fisher Scientific, Waltham, MA, USA) to discriminate between living and non-living CD4^+^ T lymphocytes. The proliferation and percentage of non-living CD4^+^ T lymphocytes were both determined by the Attune NxT Acoustic Focusing flow cytometer (Thermo Fisher Scientific, Waltham, MA, USA). The CFSE and PI dyes were excited at 488 nm, and emitted light was detected at the BL1 and BL2 channels, respectively. Unlabelled, CFSE, and PI single-labelled non-proliferating CD4^+^ T lymphocytes were used for compensation. Living and dead CD4^+^ T lymphocytes were mixed 1:1 in all three compensation controls. In total, 20,000 CD4^+^ T lymphocytes were acquired per sample. After excluding coincidence events and cell debris, non-viable CD4^+^ T lymphocytes (PI^+^) were excluded for proliferation analysis. FCS Express 7 (De Novo Software, Pasadena, CA, USA) was used for analysis. The proliferation fit algorithm from FCS Express 7 was used to determine the percentage of original CD4^+^ T lymphocytes that divided (% divided) based on

$$\frac{{\mathop \sum \nolimits_{i = 1}^{P - 1} \frac{{N^{i} }}{{2^{i} }}}}{\# of\, original \,cells} \times 100$$

with P being the total number of peaks found and N being the number of cells in a generation. Additionally, the number of divided cell generations and the number of original cells per generation were calculated as a percentage of the total number of original cells. Furthermore, the percentage of non-viable CD4^+^ T lymphocytes was determined.

### *CD4*^+^*T lymphocyte cytokine production analysis*

After five days of incubation, conditioned media from CD4^+^ T lymphocytes were harvested, centrifuged, and stored at − 80 °C. The Th lymphocyte cytokines (IL-5, IL-13, IL-2, IL-6, IL-9, IL-10, IFN-γ, TNF-α, IL-17A, IL-17F, IL-4, and IL-22) were simultaneously quantified in the conditioned media using the LEGENDplex™ Multi-Analyte Flow Assay Kit (Human Th Cytokine Panel, 12-plex, BioLegend, San Diego, CA, USA) with V-bottom plates in accordance to the manufacturer’s manual. Determined IL-6 concentrations were excluded due to the high secretion of this cytokine by hPDL-MSCs. Samples were read on the Attune Nxt Acoustic Focusing flow cytometer (Thermo Fisher Scientific, Waltham, MA, USA), setting the photomultiplier tubes (PMT) voltage in conformity with the kit instructions. After excluding debris, 3600 beads (approximately 300 beads per cytokine) were acquired. The data were analyzed using the LEGENDplex™ data analysis software (BioLegend, San Diego, CA, USA) and five-parameter logistic standard curves. The cytokine concentrations of each sample were normalized to the corresponding total CD4^+^ T lymphocyte numbers, which were determined by using Neubauer-improved cell counting chambers (NanoEnTek, Soul, South Korea).

### *Immunomediator gene expression analysis in hPDL-MSCs*

After 5 days of incubation, hPDL-MSCs’ lysis, cDNA synthesis, and the determination of *IDO-1, PTGS-2, TSG-6, CD273*, and *CD274* gene expression levels were performed by using the TaqMan Gene Expression Cells-to-CT kit (Applied Biosystems, Foster City, CA, USA) in compliance with the manufacturer’s instructions. After cell lysis, cDNA was produced by heating lysates at 37 °C for one hour, followed by 95 °C for five minutes and a cooling down to 4 °C, using the Primus 96 advanced thermocycler (PeqLab/VWR, Darmstadt, Germany). qPCR was conducted using the QuantStudio 3 device (Applied Biosystems, Foster City, CA, USA), heating the cDNA to 95 °C for ten minutes. This one-time step was followed by 50 cycles of heating the samples to 95 °C for 15 s and to 60 °C for one minute. To specifically quantify *IDO-1, PTGS-2, TSG-6, CD273,* and *CD274* associated cDNA, the following TaqMan Gene Expression Assays (Applied Biosystems, Foster City, CA, USA) were used: Hs00984148_m1 (*IDO-1*), Hs00153133_m1 (*PTGS-2*), Hs00200180_m1 (*TSG-6*), Hs00228839_m1 (*CD273*), Hs00204257_m1 (*CD274*), and Hs99999905_m1 (*GAPDH*). Glyceraldehyde-3-phosphate dehydrogenase (*GAPDH*) functioned as an internal reference. After the target genes were amplified in paired reactions, the cycle threshold (Ct) values were normalized to *GAPDH* (ΔCt), followed by the determination of the n-fold gene expression in comparison to the appropriate controls (n-fold expression = 1) by using the 2^−ΔΔCt^ formula.

### *Statistical analysis*

SPSS Statistics 26.0 (IBM, Armonk, USA) was used for statistical analysis. The Kolmogorov–Smirnov test was conducted to verify normally distributed data. Due to the non-parametric nature of the data, the Friedman Test was applied for multiple and the Wilcoxon Test for pairwise comparisons of paired groups. *P* values < 0.05 were considered as statistically significant. The data were received from at least five repetitions using hPDL-MSCs, which were isolated from at least five patients.

## Results

### In vitro*-co-culture models influence the ability of hPDL-MSCs to suppress CD4*^+^*T lymphocytes in the absence of exogenous cytokines*

Figure 1 compares the effects of hPDL-MSCs on the proliferation (Fig. 1B, D, E) and viability (Fig. 1C) of CD4^+^ T lymphocytes using three different in vitro co-culture models (Fig. 1A) without exogenous cytokines. hPDL-MSCs significantly decreased the CD4^+^ T lymphocyte proliferation (Fig. 1B) in all three in vitro co-culture models. The number of divided CD4^+^ T lymphocyte generations was reduced to four and two in the indirect and direct co-culture without insert, respectively (Fig. 1D, E). These hPDL-MSCs-based suppressive effects were significantly stronger in the direct in vitro co-culture models with and without inserts than in the indirect one (Fig. 1B). This was also confirmed by a reduced % of original CD4^+^ T lymphocytes starting from the first divided generation (G1) when directly culturing hPDL-MSCs and CD4^+^ T lymphocytes (Fig. 1D). In addition, indirectly co-cultured hPDL-MSCs showed an anti-cell-death effect, however, without any significance, whereas a direct co-culture without inserts caused a significant increase in dead CD4^+^ T lymphocytes (Fig. 1C). These data indicate that the ability of hPDL-MSCs to suppress CD4^+^ T lymphocytes depends on the used co-culture model.

### *The *in vitro* co-culture model affects the ability of hPDL-MSCs to influence cytokine production in CD4*^+^*T lymphocytes*

**Fig. 2:**
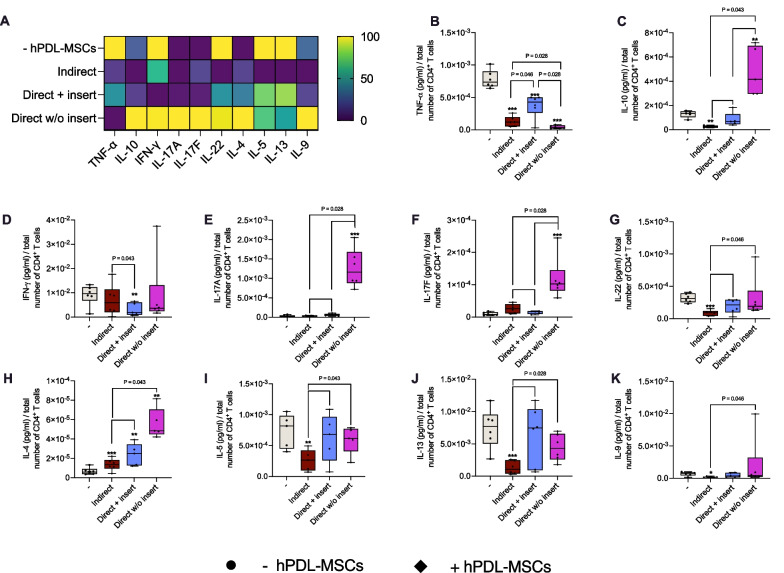
Different co-culture models cause variable effects of hPDL-MSCs on the cytokine secretion of CD4^+^ T lymphocytes. After five days of incubation, the cytokine levels were determined in conditioned media by a bead-based multiplex assay followed by flow cytometry analysis. **A** reveals a heatmap of normalized data in percentage. **B**–**K** shows the cytokine concentrations (pg/ml) normalized by the total number of CD4^+^ T lymphocytes per group. The data were obtained from five or six repetitions using hPDL-MSCs from different individuals for each repetition. In **B**–**K**, the data are displayed as box plots, also showing each individual data point. The statistical significance was evaluated by the Friedman and Wilcoxon Test for pairwise comparison. *P* values < 0.05 were statistically significant and were indicated between appropriate groups. ***P value: 0.028; **P value: 0.043: *P value: 0.046 (**B**–**K**)

Figure 2 shows the impact of hPDL-MSCs on the cytokine production in CD4^+^ T lymphocytes in different in vitro co-culture models. All three model types show various effects on the production of CD4^+^ T lymphocyte-associated cytokines in the presence of untreated hPDL-MSCs (Fig. 2A). In the indirect co-culture model, hPDL-MSCs significantly decreased the levels of TNF-α (Fig. 2B), IL-10 (Fig. 2C), IL-22 (Fig. 2G), IL-5 (Fig. 2I), IL-13 (Fig. 2J), and IL-9 (Fig. 2K). In the direct model with insert, TNF-α (Fig. 2B) and IFN-γ (Fig. 2D) were significantly reduced by hPDL-MSCs, however, to a different extent when compared to the indirect model type (Fig. 2B, D). IL-4 (Fig. 2H) levels were significantly enhanced in both co-culture models with inserts, showing a significantly higher concentration in the direct co-culture model with inserts. Directly co-culturing without inserts caused a significant reduction in TNF-α (Fig. 2B), with significantly lower levels than in the other model types. In contrast, IL-10 (Fig. 2C), IL-17A (Fig. 2E), IL-17F (Fig. 2F), and IL-4 (Fig. 2H) were significantly increased. These enhanced cytokine levels were higher compared to the other model types. Together, these data indicate that the ability of hPDL-MSCs to influence cytokine production in CD4^+^ T lymphocytes depends on the in vitro co-culture model.

### *The type of *in vitro* co-culture model dictates the effects of IL-1β-treated hPDL-MSCs on the CD4*^+^*T lymphocyte proliferation and viability*

**Fig. 3:**
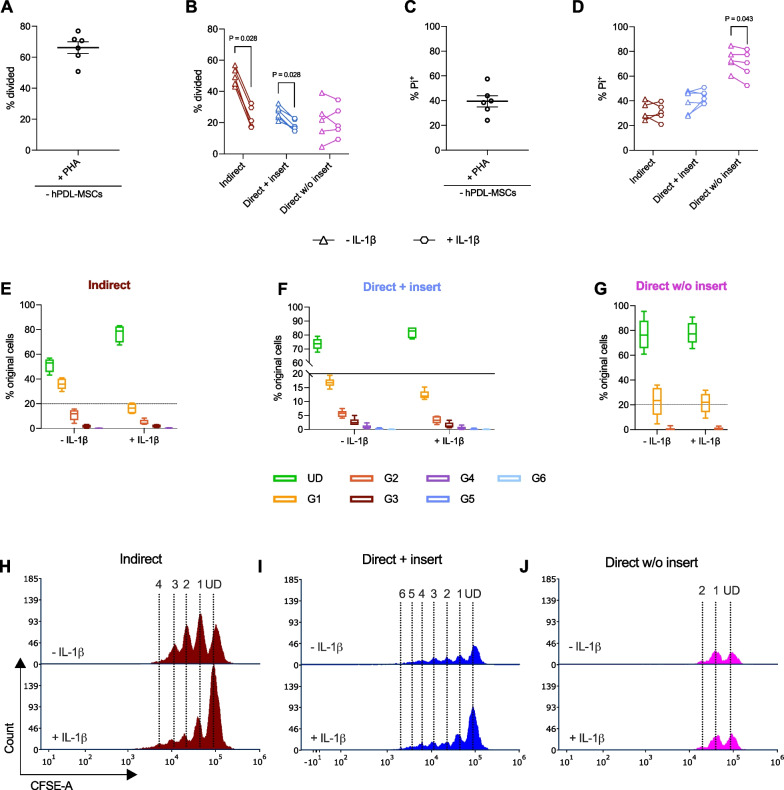
The influence of IL-1β-treated hPDL-MSCs on CD4^+^ T lymphocyte proliferation and viability depends on the co-culture model. After five days of incubation of CD4^+^ T lymphocytes with untreated or IL-1β-treated hPDL-MSCs, the proliferation and viability were measured by flow cytometry, based on CFSE (**A**, **B**, **E**–**J**) and PI (**C**, **D**) staining. **A** and **B** reveal the percentage of original CD4^+^ T lymphocytes that have divided at least once, whereas **C** and **D** show the percentage of non-viable CD4^+^ T lymphocytes. The number of original CD4^+^ T lymphocytes for the undivided (UD) and each divided (G1–G6) generation (**E**–**G**) is presented as a percent of the total number of original CD4^+^ T lymphocytes. The absolute numbers of CD4^+^ T lymphocytes in each generation (UD, and G1–G6) are shown in representative histograms (**H**–**J**). For the proliferation analysis, the data were obtained from six (indirect and direct + insert) and five (direct w/o insert) repetitions, whereas the viability was determined from five (indirect and direct w/o insert) and six (direct + insert) repetitions. hPDL-MSCs from different individuals were used for each repetition. In **A**–**D**, mean values measured in each experimental repetition are connected with lines. In **E**–**G**, data are presented as box-whisker plots showing the minimum and maximum values. The statistical significance was evaluated by the Friedman Test, followed by the Wilcoxon Test for pairwise comparison. *P* values < 0.05 were statistically significant and were indicated between appropriate groups (**B**, **D**)

Figure 3 exhibits the effects of IL-1β-triggered hPDL-MSCs on the proliferation (Fig. 3A, B, E–J) and viability (Fig. 3C, D) of CD4^+^ T lymphocytes using different in vitro co-culture models. The presence of IL-1β significantly strengthened the inhibitory effects of hPDL-MSCs toward PHA-activated CD4^+^ T lymphocyte proliferation when co-cultured indirectly and directly with insert (Fig. 3A, B). This was also indicated by a reduced % of original CD4^+^ T lymphocytes starting from the first divided generation G1 (Fig. 3E–J). The direct co-culture without inserts showed no apparent effect of hPDL-MSCs on the CD4^+^ T lymphocyte proliferation in the presence of IL-1β (Fig. 3A), without changes in the % of original CD4^+^ T lymphocytes per generation (Fig. 3G) and the number of divided generations (Fig. 3J). However, the hPDL-MSCs-mediated cell-death-inducing effect (Fig. 3C, D) was significantly counteracted by IL-1β-treated hPDL-MSCs in the direct co-culture without inserts (Fig. 3D). These data indicate that the ability of IL-1β-treated hPDL-MSCs to affect CD4^+^ T lymphocyte proliferation and viability depends on the used co-culture model.

### *The type of *in vitro* co-culture model dictates the effects of IL-1β-treated hPDL-MSCs on cytokine production in CD4*^+^*T lymphocytes*

**Fig. 4:**
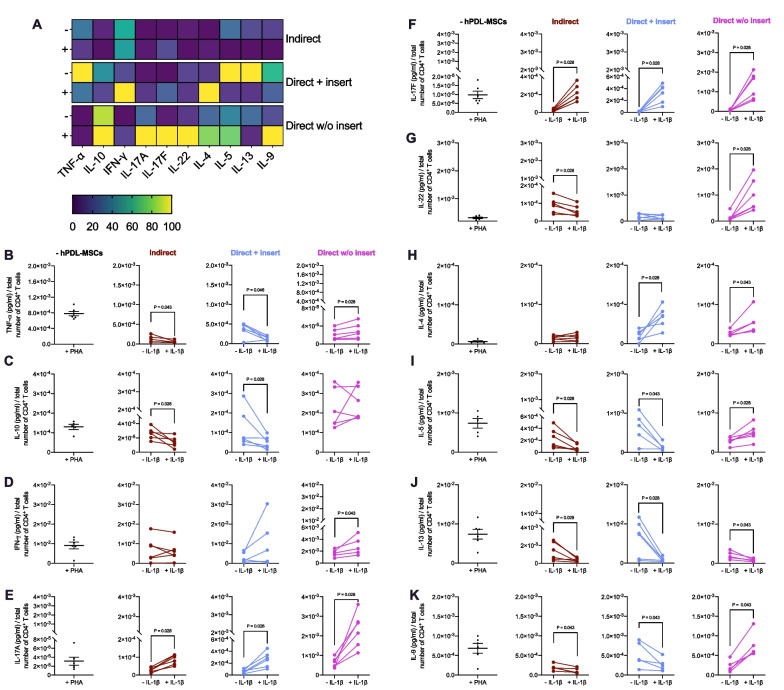
Co-culture models cause variable effects of IL-1β-treated hPDL-MSCs on the cytokine secretion of CD4^+^ T lymphocytes. After five days of incubation of CD4^+^ T lymphocytes with untreated or IL-1β-treated hPDL-MSCs, the cytokine levels were determined in conditioned media by a bead-based multiplex assay followed by flow cytometry analysis. **A** reveals a heatmap of normalized data in percentage. **B**–**K** shows the cytokine concentrations (pg/ml) normalized by the total number of CD4^+^ T lymphocytes per group. The data were obtained from five or six repetitions using hPDL-MSCs from different individuals for each repetition. In **B**–**K**, mean values measured in each experimental repetition are connected with lines. The statistical significance was evaluated by the Friedman and Wilcoxon Test for pairwise comparison. *P* values < 0.05 were statistically significant and were indicated between appropriate groups (**B**–**K**)

Figure 4 demonstrates the effects of IL-1β-treated hPDL-MSCs on cytokine production in CD4^+^ T lymphocytes using different in vitro co-culture models. The indirect and direct co-culture models with insert caused a similar effect of IL-1β-treated hPDL-MSCs on the cytokine secretion profile of CD4^+^ T lymphocytes (Fig. 4A–K): TNF-α (Fig. 4B), IL-10 (Fig. 4C), IL-5 (Fig. 4I), IL-13 (Fig. 4J), and IL-9 (Fig. 4K) were significantly reduced, whereas IL-17A (Fig. 4E), and IL-17F (Fig. 4F) were significantly upregulated in the presence of IL-1β-treated hPDL-MSCs; IL-22 (Fig. 4G) and IL-4 (Fig. 4H) were significantly up- and down-regulated in the indirect and direct co-culture model with insert, respectively, whereas IFN-γ (Fig. 4A, D) was not significantly changed in both co-culture models.

In contrast, direct co-culturing CD4^+^ T lymphocytes with IL-1β-treated hPDL-MSCs significantly triggered increased levels of all investigated cytokines (Fig. 4A–B, D–I, K), except IL-13 (Fig. 4J) showing a significant decline. No significant changes were observed for IL-10 (Fig. 4A, C). These data indicate that the ability of IL-1β-treated hPDL-MSCs to influence cytokine production in CD4^+^ T lymphocytes depends on the in vitro co-culture model.

### *The type of *in vitro* co-culture model dictates the effects of TNF-α-treated hPDL-MCs on the CD4*^+^*T lymphocyte proliferation and viability*

**Fig. 5:**
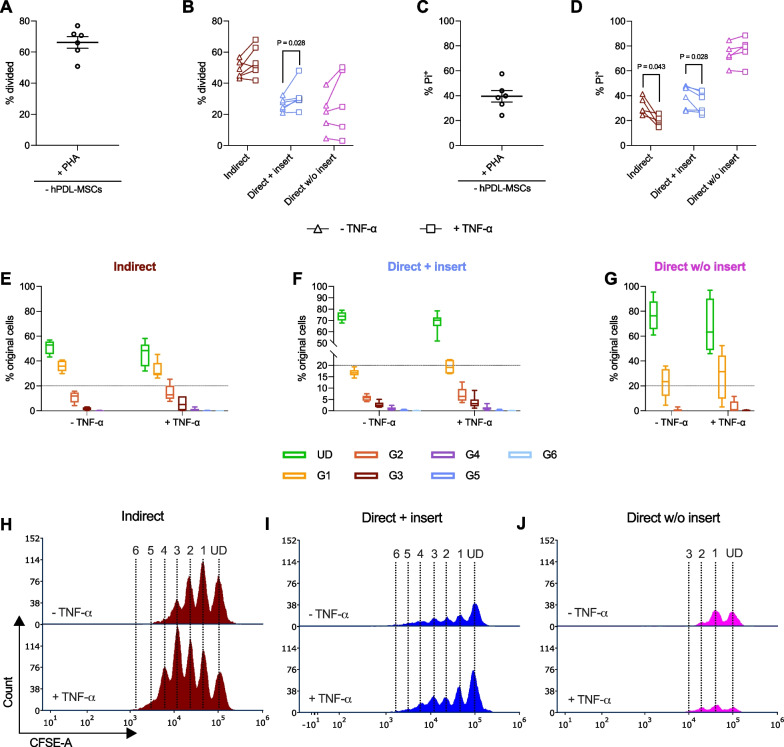
The influence of TNF-α-treated hPDL-MSCs on CD4^+^ T lymphocyte proliferation and viability depends on the co-culture model. After five days of incubation of CD4^+^ T lymphocytes with untreated or TNF-α-treated hPDL-MSCs, the proliferation and viability were measured by flow cytometry, based on CFSE (**A**, **B**, **E**–**J**) and PI (**C**, **D**) staining. **A** and **B** reveal the percentage of original CD4^+^ T lymphocytes that have divided, whereas **C** and **D**  show the percentage of non-viable CD4^+^ T lymphocytes. The number of original CD4^+^ T lymphocytes for the undivided (UD) and each divided (G1-G6) generation (**E**–**G**) is presented as a percent of the total number of original CD4^+^ T lymphocytes. The number of CD4^+^ T lymphocytes in each generation (UD, and G1–G6) are shown in representative histograms (**H**–**J**). For the proliferation analysis, the data were obtained from six (indirect and direct + insert) and five (direct w/o insert) repetitions, whereas the viability was determined from five (indirect and direct w/o insert) and six (direct + insert) repetitions. hPDL-MSCs from different individuals were used for each repetition. In **A**–**D**, mean values measured in each experimental repetition are connected with lines. In **E**–**G**, data are presented as box-whisker plots showing the minimum and maximum values. The statistical significance was evaluated by the Friedman Test and the Wilcoxon Test for pairwise comparison. *P* values < 0.05 were statistically significant and were indicated between appropriate groups (**B**, **D**)

Figure 5 reveals the effects of TNF-α-triggered hPDL-MSCs on the CD4^+^ T lymphocyte proliferation (Fig. 5A, B, E–J) and viability (Fig. 5C, D) using different in vitro co-culture models. The presence of TNF-α significantly counteracted the hPDL-MSCs-mediated inhibition of PHA-activated CD4^+^ T lymphocytes when co-cultured directly with inserts (Fig. 5A, B). This was also indicated by an increased % of original CD4^+^ T lymphocytes in the dividing generations G1-G3 (Fig. 5F, I). The indirect and direct co-culture model without inserts showed no clear effect of TNF-α-treated hPDL-MSCs on the CD4^+^ T lymphocyte proliferation (Fig. 5B), without remarkable changes in the % of original CD4^+^ T lymphocytes per generation (Fig. 5E, G). However, TNF-α-stimulated hPDL-MSCs caused an increase in the number of divided CD4^+^ T lymphocyte generations from four to six and from two to three dividing generations in the indirect (Fig. 5E, H) and direct without insert (Fig. 5G, J) co-culture model, respectively. In addition, TNF-α-treated hPDL-MSCs significantly reduced the percentage of PI^+^ CD4^+^ T lymphocytes in the indirect and direct co-culture systems with inserts (Fig. 5C, D). These data indicate that the ability of TNF-α-treated hPDL-MSCs to affect CD4^+^ T lymphocyte proliferation and viability depends on the co-culture model.

### *The type of *in vitro* co-culture model dictates the effects of TNF-α-treated hPDL-MSCs on cytokine production in CD4*^+^*T lymphocytes*

**Fig. 6:**
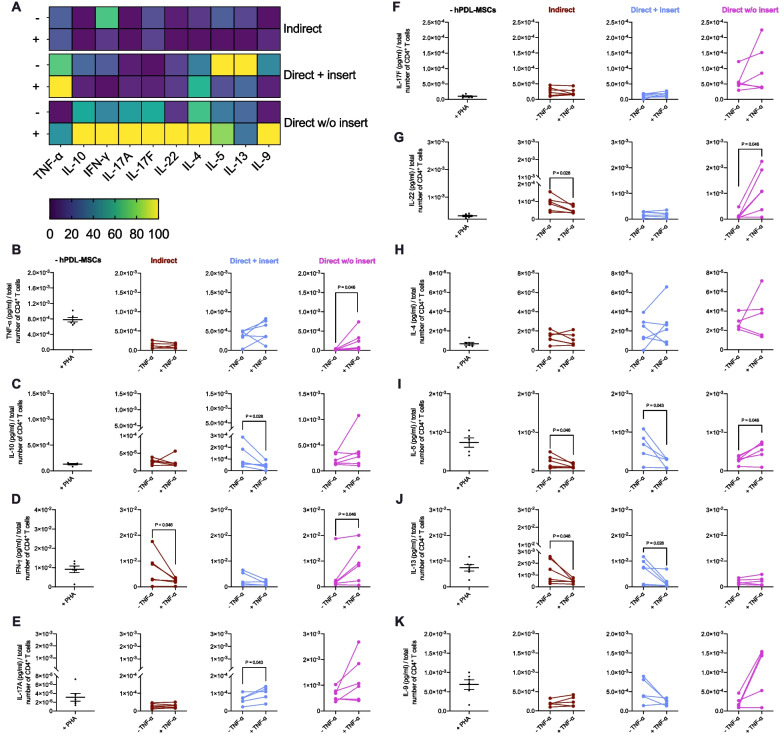
Co-culture models cause variable effects of TNF-α-triggered hPDL-MSCs on the cytokine secretion of CD4^+^ T lymphocytes. After five days of incubation of CD4^+^ T lymphocytes with untreated and TNF-α-treated hPDL-MSCs, the cytokine levels were determined in condition media by a bead-based multiplex assay followed by flow cytometry analysis. **A** reveals a heatmap of normalized data in percentage. **B**–**K** show the cytokine concentrations (pg/ml) normalized by the total number of CD4^+^ T lymphocytes per group. The data were obtained from five or six repetitions using hPDL-MSCs from different individuals for each repetition. In **B**–**K**, mean values measured in each experimental repetition are connected with lines. The statistical significance was evaluated by the Friedman and Wilcoxon Test for pairwise comparison. *P* values < 0.05 were statistically significant and were indicated between appropriate groups **B**–**K**

Figure 6 demonstrates the effects of TNF-α-treated hPDL-MSCs on cytokine production in CD4^+^ T lymphocytes using different in vitro co-culture models. In the indirect co-culture model (Fig. 6A), a significant decrease in IFN-γ (Fig. 6D), IL-22 (Fig. 6G), IL-5 (Fig. 6I), and IL-13 (Fig. 6J) levels was observed in the presence of TNF-α-stimulated hPDL-MSCs. A significant reduction in IL-10 (Fig. 6C), IL-5 (Fig. 6I), and IL-13 (Fig. 6J) levels was caused by TNF-α-treated hPDL-MSCs directly co-cultured with CD4^+^ T lymphocytes with insert, whereas IL-17A (Fig. 6E) was significantly enhanced. A significant gain in TNF-α (Fig. 6B), IFN-γ (Fig. 6D), IL-22 (Fig. 6G), and IL-5 (Fig. 6I) levels was caused by directly co-cultured TNF-α-treated hPDL-MSCs without insert. These data suggest that the in vitro co-culture model affects the ability of TNF-α-treated hPDL-MSCs to change the cytokine production in CD4^+^ T lymphocytes.

### *The *in vitro* co-culture model type influences the immunomediator gene expression levels in hPDL-MSCs*

**Fig. 7:**
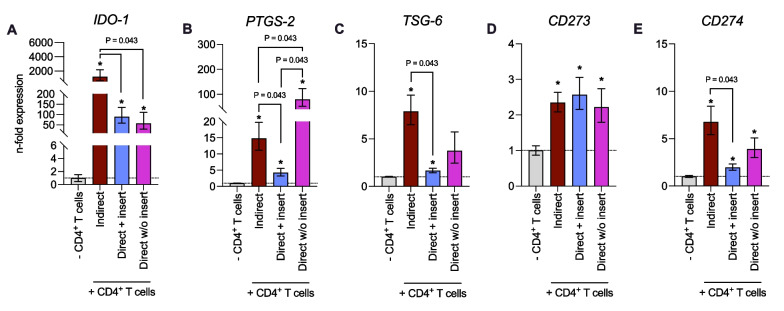
The immunomediator gene expression in hPDL-MSCs alters depending on the co-culture model. After five days of incubation, *IDO-1* (**A**), *PTGS-2* (**B**), *TSG-6* (**C**), *CD273* (**D**), and *CD274* (**E**) gene expression levels were determined in co-cultured hPDL-MSCs using qPCR. The x-axes show the n-fold expression compared to hPDL-MSCs cultured without CD4^+^ T lymphocytes (n-fold expression = 1). *GAPDH* served as a reference gene. The data were obtained from five repetitions using hPDL-MSCs from different individuals for each iteration and two technical replicates per group. The data are presented as mean + /– S.E.M.. The Friedman and Wilcoxon tests for pairwise comparison were used to identify statistical significance. *P* values < 0.05 were statistically significant and were indicated between appropriate groups. *P value < 0.05 compared to the hPDL-MSCs without CD4^+^ T-lymphocytes

Figure 7 shows immunomediator gene expression levels in hPDL-MSCs at the end of co-culture experiments in different model types. Compared to the hPDL-MSCs monoculture, the gene expression levels of all investigated immunomediators were significantly increased in hPDL-MSCs regardless of the used in vitro co-culture model (Fig. 7A–E), except *TSG-6* in hPDL-MSCs co-cultured directly without inserts (Fig. 7C). *IDO-1* (Fig. 7A), *TSG-6* (Fig. 7C), and *CD274* (Fig. 7E) gene expression levels in hPDL-MSCs were reduced in the two direct co-culture models compared to the indirect one, showing significant reduction in the direct co-culture model with insert for *IDO-1*, *TSG-6*, and *CD274*, and a significant decrease in the direct co-culture model without insert for *IDO-1*. Additionally, *PTGS-2* gene expression (Fig. 7B) was significantly reduced in hPDL-MSCs when cultured directly with insert compared to the other two models. In contrast, the direct co-culture model without insert caused a significant increase in *PTGS-2* gene expression levels in hPDL-MSCs. No differences in gene expression levels between the various in vitro co-culture models were observed for *CD273* (Fig. 7D). These data indicate that the type of the co-culture models impacts the immunomediator gene expression in hPDL-MSCs.

### *The gene expression of membrane-bound immunomediators in hPDL-MSCs is differently influenced by the *in vitro* co-culture model type in the presence of exogenous IL-1β*

**Fig. 8:**
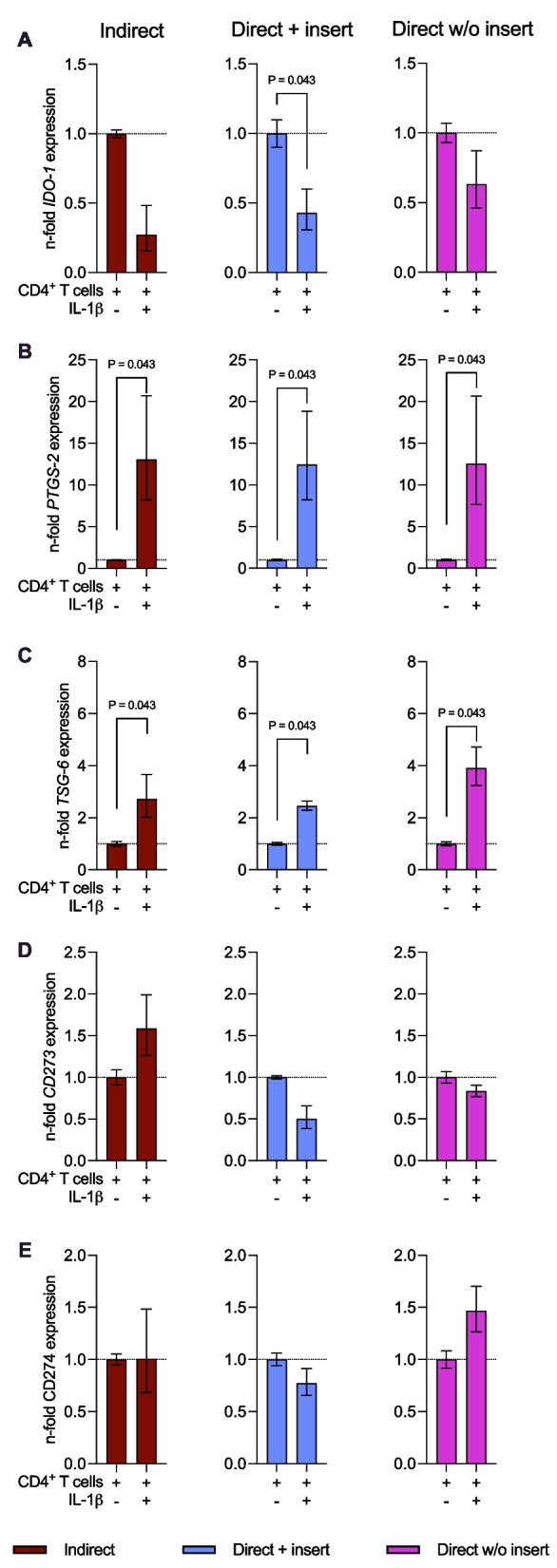
IL-1β-triggered immunomediator gene expression in hPDL-MSCs is variably influenced by the different co-culture models. After five days of incubation, *IDO-1* (**A**), *PTGS-2* (**B**), *TSG-6* (**C**), *CD273* (**D**), and *CD274* (**E**) gene expression levels were determined in hPDL-MSCs co-cultured with CD4^+^ T lymphocytes indirectly, directly with insert, or directly without insert. After performing qPCR, the n-fold expression levels were calculated and compared to the appropriate controls within the different co-culture models. hPDL-MSCs co-cultured with CD4^+^ T lymphocytes in the absence of exogenous IL-1β served as control (n-fold expression levels = 1). *GAPDH* was used as a reference gene. The data were obtained from five repetitions using hPDL-MSCs from different individuals for each iteration and two technical replicates per group. The data are presented as mean + /– S.E.M. The Friedman and Wilcoxon Tests for pairwise comparison were used to demonstrate statistical significance. *P* values < 0.05 were statistically significant and were indicated between appropriate groups

Figure 8 displays the immunomediator gene expression in IL-1β-treated hPDL-MSCs when co-cultured with CD4^+^ T lymphocytes indirectly, directly with insert, or directly without insert. The gene expression levels of soluble immunomediators (Fig. 8A–C) were influenced by exogenous IL-1β regardless of the co-culture model: *IDO-1* gene expression (Fig. 8A) was decreased in the presence of exogenous IL-1β, with a significant reduction for the direct co-culture model with insert. In contrast, *PTGS-2* (Fig. 8B) and *TSG-6* (Fig. 8C) were significantly upregulated in IL-1β-treated hPDL-MSCs.

IL-1β caused an increase and decrease of the *CD273* (Fig. 8D) in hPDL-MSCs when indirectly and directly co-cultured with CD4^+^ T lymphocytes, respectively. *CD274* gene expression (Fig. 8E) was decreased and increased in IL-1β-treated hPDL-MSCs directly co-cultured with CD4^+^ T lymphocytes with and without inserts, respectively. No effect on *CD274* gene expression was observed in the indirect co-culture model. Although these data indicate some tendencies, significant differences were not observed for the membrane-bound immunomediator gene expression levels. Together, these data suggest that membrane-bound immunomediators, but not soluble ones, are differently influenced in hPDL-MSCs by the in vitro co-culture model type in the presence of exogenous IL-1β.

### *The gene expression of soluble and membrane-bound immunomediators in hPDL-MSCs is differently influenced by the *in vitro* co-culture model type in the presence of exogenous TNF-α*

**Fig. 9:**
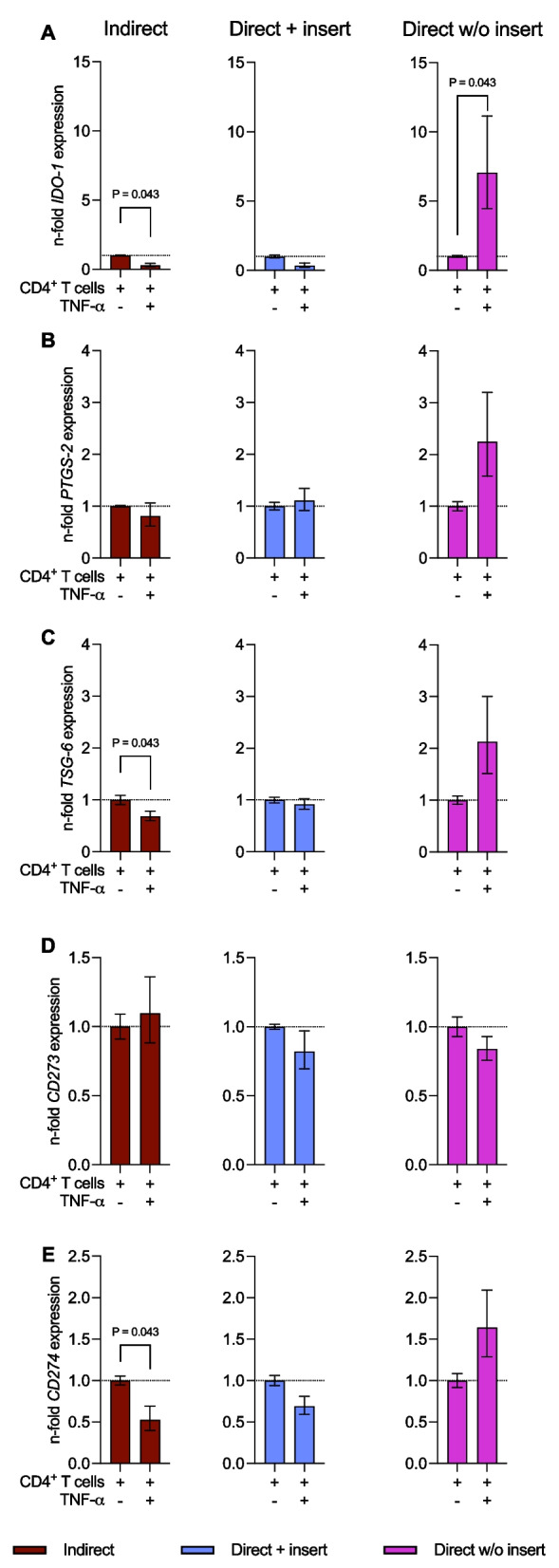
The different co-culture models variably influence the TNF-α-triggered immunomediator gene expression in hPDL-MSCs. After five days of incubation, *IDO-1* (**A**), *PTGS-2* (**B**), *TSG-6* (**C**), *CD273* (**D**), and *CD274* (**E**) gene expression levels were determined in hPDL-MSCs co-cultured with CD4^+^ T lymphocytes indirectly, directly with insert, or directly without insert. After performing qPCR, the n-fold expression levels were calculated and compared to the appropriate controls within the different co-culture models. hPDL-MSCs co-cultured with CD4^+^ T lymphocytes without exogenous TNF-α served as control (n-fold expression = 1). *GAPDH* was used as a reference gene. The data were obtained from five repetitions using hPDL-MSCs from different individuals for each iteration and two technical replicates per group. The data are presented as mean + /– S.E.M. The Friedman and Wilcoxon Tests for pairwise comparison were used to demonstrate statistical significance. *P* values < 0.05 were statistically significant and were indicated between appropriate groups

Figure 9 shows the immunomediator gene expression in TNF-α-treated hPDL-MSCs when co-cultured with CD4^+^ T lymphocytes indirectly, directly with insert, or directly without insert. The gene expression levels of the soluble immunomediators (Fig. 9A–C) were reduced in TNF-α-stimulated hPDL-MSCs when co-cultured indirectly with CD4^+^ T lymphocytes, showing significant reductions for *IDO-1* (Fig. 9A) and *TSG-6* (Fig. 9C). In the direct co-culture with insert, TNF-α caused a slight decrease in *IDO-1* (Fig. 9A) and *TSG-6* (Fig. 9C) gene expression, whereas *PTGS-2* (Fig. 9B) was negligibly increased. In the directly co-cultured hPDL-MSCs without insert, the gene expression levels of soluble immunomediators (Fig. 9A–C) were increased in the presence of TNF-α, showing a significant enhancement for *IDO-1* (Fig. 9A). *CD273* gene expression levels (Fig. 9D) were slightly increased and decreased in TNF-α-treated hPDL-MSCs when indirectly and directly co-cultured with CD4^+^ T lymphocytes, respectively. In the indirect and direct co-culture type with insert, TNF-α caused a reduction in *CD274* gene expression in hPDL-MSCs, whereas the direct co-culture model without inserts increased *CD274 expression* (Fig. 9E). Significant changes in the gene expression levels of membrane-bound immunomediators were only observed for *CD274* in indirectly co-cultured, TNF-α-treated hPDL-MSCs. These data indicate that the gene expression of soluble and membrane-bound immunomediators in hPDL-MSCs is differently influenced by the in vitro co-culture model type in the presence of exogenous TNF-α.

## Discussion

In vitro [4] and animal studies [8–10, 23, 24] provide promising results for using MSCs, including hPDL-MSCs [25], as a therapeutic tool for degenerative and inflammatory diseases. Although the European Medical Agency already approved the first MSCs’ containing products [26] in Europe, clinical trials mainly lead to sobering outcomes [27, 28]. Since the therapeutic potential of MSCs is primarily based on their immunomodulatory abilities [11], there is a great effort for their enhancement. The mechanisms of MSCs-mediated immunomodulation are versatile, and various co-culture models have been widely used in hPDL-MSCs’ research [14, 16–19]. However, the comparison of these studies is aggravated by differences in the co-culture-associated conditions and parameters, including the usage of PBMCs or pure CD4^+^ T lymphocytes. Hence, to better understand the immunomodulatory effects of hPDL-MSCs, studies are necessary to compare the various co-culture models under controlled and comparable conditions. Therefore, in this study, we investigated the effects of direct and indirect co-culture models on CD4^+^ T lymphocytes under standardized conditions to reduce confounding effects. We used only models, in which a bidirectional interaction between hPDL-MSCs and CD4^+^ T lymphocytes takes place [14, 16–19], and therefore, the use of hPDL-MSCs’ conditioned media for CD4^+^ T lymphocytes treatment was not considered [16].

This study revealed that hPDL-MSCs affect CD4^+^ T lymphocyte proliferation, variability, and cytokine secretion depending on the co-culture model. The distinct co-culture models also display variability in gene expression levels of the immunomodulatory factors *IDO-1*, *PTGS-2*, *TSG-6*, *CD273*, and *CD274* in hPDL-MSCs. Together, these data indicate that the co-culture models differently impact the reciprocal interaction between hPDL-MSCs and CD4^+^ T lymphocytes, suggesting that the paracrine and direct cell-to-cell contact immunomodulatory mechanisms are responsible for the detected differences.

In the absence of exogenous cytokines, hPDL-MSCs caused suppression of CD4^+^ T lymphocyte proliferation in all the co-culture models, which is in accordance with previous studies [14, 16–19]. However, the degree of suppression was significantly higher in the two direct co-culture models than the indirect one. Additionally, in the direct co-culture model without insert, only two divided CD4^+^ T lymphocyte generations were detected in the presence of hPDL-MSCs, indicating a complete loss of the proliferation potential. This contrasts the indirect and direct co-culture models, in which up to six divided generations were detected.

Inhibition of CD4^+^ T lymphocyte proliferation in the presence of hPDL-MSCs in the indirect model was not accompanied by increased cell death. Moreover, a slight reduction in the number of dead CD4^+^ T lymphocytes in the presence of indirectly co-cultured hPDL-MSCs was observed, which agrees with our previous study [14]. In contrast, further inhibition of CD4^+^ T lymphocyte proliferation in the direct co-culture model without insert was accompanied by a substantially increased cell death. Interestingly, an additional inhibition of CD4^+^ T lymphocyte proliferation in the direct model with inserts was not accompanied by an increased cell death. Hence, the following conclusion could be made: First, in the indirect model, in which the interaction between two cell types is mediated by the paracrine mechanisms only, hPDL-MSCs-mediated suppression of CD4^+^ T lymphocyte proliferation is not based on inducing CD4^+^ T lymphocyte death. Second, limited interaction between two cell types through a 0.4 μm porous membrane has some additional inhibitory effects on CD4^+^ T lymphocytes, which is also not associated with their cell death. Third, in the direct model without inserts, where the direct interaction of CD4^+^ T lymphocytes with hPDL-MSCs is unlimited, the hPDL-MSCs-induced inhibition of the CD4^+^ T lymphocyte proliferation is due to the induction of CD4^+^ T lymphocyte death rather than arresting them in the G0/G1 cell cycle phase [3].

Induction of CD4^+^ T lymphocyte apoptosis by hPDL-MSCs upon direct contact could be associated with increased *CD273* and *CD274* expression levels in hPDL-MSCs, which both induce T lymphocyte apoptosis [29]. Although these enhanced levels were observed in all three co-culture settings, binding of PD-L2 and PD-L1 transmembrane proteins, which are coded by these genes, to their receptor PD-1 on the T lymphocyte surface is only possible in the direct co-culture model triggering CD4^+^ T lymphocyte death [29]. Besides, some other mechanisms are also possible. One study also revealed the induction of cell death in T lymphocytes by directly co-culturing dental pulp-derived MSCs via the transmembrane protein Fas ligand, which is an apoptosis inducer [30]. In contrast, previous studies revealed that the suppressive effects of MSCs on T lymphocyte proliferation are cell death independent in a direct co-culture setting [31, 32]. Using MSCs from the bone marrow [23, 24] isolated from animals [24] may contribute to these inconsistent data.

The production of various cytokines by PHA-activated CD4^+^ T lymphocytes was also substantially affected by hPDL-MSCs, and this effect was dependent on the experimental setting. Compared to the CD4^+^ T lymphocyte monoculture, indirect co-culture of CD4^+^ T lymphocytes with hPDL-MSCs resulted in significantly lower TNF-α, IL-5, IL-9, IL-10, IL-13, and IL-22 levels. Most of these cytokines are produced by either Th1 or Th2 subsets, and therefore, it seems that hPDL-MSCs inhibit Th1 and Th2 responses, and the paracrine mechanisms are responsible for this effect. This is partly in accordance with Aggarwal et al. [33], who also observed an inhibitory effect of bone marrow-derived MSCs on the Th1 response but an increase in Th2-associated cytokines. This may be explained using a direct co-culture model.The presence of contact between CD4^+^ T lymphocytes and hPDL-MSCs further impacted cytokine production, and the most striking effect was observed when this contact was not limited by the porous membrane. The most conspicuous differences were a higher production of IL-4, IL-10, and IL-17 family cytokines and a further inhibition of TNF-α production. These data imply that direct cell contact might promote the Th17 response. A similar effect has already been reported for bone marrow MSCs [34]. Increased production of IL-4 and IL-10 might suggest that direct cell-to-cell contact also promotes the Th2 response. This assumption is also enforced by significantly higher production of other Th2 cytokines, particularly IL-5, IL-9, and IL-13, in the direct co-culture model with insert compared to the indirect co-culture model. Thus, the Th2 response seems to be inhibited by the paracrine mechanisms and promoted by the direct cell-to-cell contact.

In the next step, we tested how boosting the immunomodulatory properties of hPDL-MSCs by adding exogenous cytokines influenced their effect on CD4^+^ T lymphocytes in different co-culture models. Our data revealed that IL-1β substantially enhanced the ability of hPDL-MSCs to suppress CD4^+^ T lymphocyte proliferation in the indirect co-culture model, which agrees with our previous study [14]. A similar ability, although to a lesser extent, was observed for the direct model with insert. In contrast, no additional effect of IL-1β treatment was observed in the direct model without insert. These observations suggest that IL-1β might stimulate primarily the production of the soluble mediators, affecting the proliferation of CD4^+^ T lymphocytes. qPCR analysis showed that IL-1β enhanced the gene expression of *TSG-6* and *PTGS-2*, which is responsible for the production of PGE_2_. These proteins might be responsible for the observed effects. Particularly, PGE_2_ was verified in a previous study to be responsible for the inhibitory effect of MSCs on the proliferation of lymphocytes [35], and for reducing the Th1 response [33]. Furthermore, IL-1β slightly diminished the pro-apoptotic effect of hPDL-MSCs on the CD4^+^ T lymphocytes in the direct model without insert. However, this effect was rather small, and the cell death rate remained high in comparison with the two other models.

The treatment of hPDL-MSCs with IL-1β also influenced their effect on the cytokine production by CD4^+^ T lymphocytes. For some cytokines, this effect was qualitatively the same in all models. Particularly, the presence of IL-1β resulted in an increased production of IL-17A, and IL-17F and a decreased production of IL-9, which suggests that IL-1β might affect some paracrine mechanisms in hPDL-MSCs, influencing the production of these cytokines. For other cytokines, the effect of IL-1β was qualitatively dependent on the co-culture model. The most striking differences were observed between the direct model without inserts and the two other models. This observation suggests that IL-1β activates some factors, participating in cell-to-cell contact and influencing the differentiation of CD4^+^ T lymphocytes.

The treatment of hPDL-MSCs with TNF-α also influenced their immunomodulatory effect on CD4^+^ T lymphocytes, depending on the co-culture model. However, these effects were less pronounced and qualitatively different from those induced by IL-1β. Particularly, the presence of TNF-α slightly abolished the inhibitory effect of hPDL-MSCs in the direct model with inserts. A similar tendency was observed in the indirect model. In both models, TNF-α treatment also exhibited some anti-cell death effects toward CD4^+^ T cells. In contrast, in the direct model without inserts, neither proliferation nor apoptosis of CD4^+^ T lymphocytes was affected by TNF-α. The treatment of hPDL-MSCs with TNF-α also affected the production of cytokines by co-cultured CD4^+^ T lymphocytes, and this effect was dependent on the co-culture model. The most striking effects were observed in the direct model without inserts. In two other models, the effects were rather minimal and were always opposite to those observed in the direct model without inserts. This observation implies that TNF-α activates some mechanisms in hPDL-MSCs, regulating cytokine production by CD4^+^ T lymphocytes through direct cell-to-cell contact. The gene expression data of our study showed no effect of TNF-α on the expression of *CD273* and *CD274* genes in hPDL-MSCs. Therefore, some other mechanisms, which are still to be identified, should be involved.

Interestingly, the response to IL-1β or TNF-α treatment regarding CD4^+^ T lymphocyte proliferation and cytokine production was quite similar in the indirect and direct model with insert than in the direct model without insert. This observation suggests that the contact between two cell types in the direct co-culture model with insert is still limited. Under these conditions, cell interaction occurs mainly through the paracrine mechanisms. The data within the different co-culture models were partially witnessed in previous studies. Whereas the observed effects within the indirect co-culture model were confirmed by our previous study [14], Ren et al. [36] and Hemeda et al. [37] also observed no clear impact of exogenous TNF-α and IL-1β when directly co-culturing MSCs and T lymphocytes without insert. To the best of our knowledge, no previous study has used a direct co-culture model with an insert to investigate cytokine-primed MSCs' influence on T lymphocytes.

Our study primarily focused on the reciprocal interaction between hPDL-MSCs and allogeneic CD4^+^ T lymphocytes in vitro and did not provide any clinical insights at first glance. Nevertheless, it is worth attempting to extrapolate our findings to the clinical situation, which is quite challenging because there is no unified clinical protocol for MSC treatment. The delivery of MSCs in clinics depends on the clinical situation; they can be injected intravenously or directly into the tissue [38]. The mechanisms by which injected MSCs execute their therapeutic effect are versatile and depend on the application. It is plausible to assume that different immune cells are affected by MSCs under various conditions. Our study found that the effect of hPDL-MSCS on allogeneic CD4^+^ T lymphocytes depends on the experimental conditions, and the direct contact between these cell types drastically increased the percentage of dead CD4^+^ T lymphocytes. This effect of MSCs might be especially important for the treatment of various autoimmune diseases, which are characterized by abnormal T lymphocyte immunity and impaired apoptosis [39, 40].

In summary, these data suggest that the effects of cytokine-treated hPDL-MSCs on CD4^+^ T lymphocyte proliferation depend on the combination of the present exogenous cytokine and the co-culture model. The monitored immunomodulatory mechanisms may partially explain these effects. In the presence of exogenous IL-1β, most immunomediators are upregulated, regardless of the used model, which may be responsible for the observed hPDL-MSCs-mediated inhibitory effects in the indirect and direct co-culture with insert. In contrast, exogenous TNF-α negatively affected the immunomediator gene expression in the models with an insert, which may explain the hPDL-MSCs-mediated immunostimulating effects in these models.

To the best of our knowledge, this is the first study that directly compares the secretion of CD4^+^ T lymphocyte subset-specific cytokines between different co-culture models. Nevertheless, our results partly agree with previous studies investigating CD4^+^ T lymphocyte-associated cytokine secretion in direct [17, 41] or indirect [42] co-culture models with MSCs from various tissues. The discrepancies, which are related to single cytokine secretions, may be explained by the lack of priming MSCs with exogenous cytokines during co-culture, using different T lymphocyte-to-MSCs ratios, various incubation times, PBMCs instead of pure CD4^+^ T lymphocyte population, and different CD4^+^ T lymphocyte activation stimuli [17, 41, 42].

## Conclusion

In conclusion, this study indicates a significant influence of the co-culture model on the outcomes concerning CD4^+^ T lymphocyte proliferation, viability, and cytokine secretion. These variable effects are observed with untreated and cytokine-treated hPDL-MSCs and may be caused by the variability of the immunomodulatory mechanisms in the differently co-cultured hPDL-MSCs. Altogether, these differences may have their origin in the fact that the co-culture models allow different bi-directional interactions (paracrine + direct cell-to-cell contact or only paracrine) between hPDL-MSCs and CD4^+^ T lymphocytes. 

Although the direct co-culture models are most suitable to mimic the in vivo situation, the indirect co-culture model allows to discriminate between paracrine and direct cell-to-cell contact immunomodulatory mechanisms. Hence, conducting the different co-culture models in parallel and directly comparing the outcomes would provide information about the involved immunomodulatory mechanisms. Therefore, conducting the different co-culture models in parallel should become good scientific practice. Additionally, the observed differences between the two direct co-culture models emphasize that a detailed description of how to co-culture MSCs and immune cells (with or without transwell insert) is essential to be included in future studies.

### Supplementary Information

Supplementary Material 1.

Supplementary Material 2.

Supplementary Material 3.

Supplementary Material 4.

Supplementary Material 5.

## Data Availability

The gene expression raw data are included in this published article as supplementary information files. The other datasets used and analyzed during the current study are available from the corresponding author upon reasonable request.

## Abbreviations

BSA: Bovine serum albumin
CFSE: Carboxyfluorescein succinimidyl ester
Ct: Cycle threshold
DMEM: Dulbecco’s modified Eagle’s medium
FBS: Fetal bovine serum
GAPDH: Glyceraldehyde-3-phosphate dehydrogenase
hPDL-MSCs: Periodontal ligament-derived mesenchymal stromal cells
IDO-1: Indoleamine-2,3-dioxygenase-1
IFN-γ: Interferon-γ
IL: Interleukin
MSCs: Mesenchymal stromal cells
PBMC: Peripheral blood mononuclear cells
PBS: Phosphate-buffered salin
PD-L1: Programmed cell death ligand 1
PDL: Periodontal ligament
PGE_2_: Prostaglandin E_2_
PHA-L: Phytohemagglutinin-L
PI: Propidium iodide
PMT: Photomultiplier tubes
qPCR: Quantitative polymerase chain reaction
S.E.M.: Standard error of the mean
TC: Transwell inserts
TNF-α: Tumor necrosis factor-α
T_regs_: Regulatory CD4+ T lymphocytes
TSG-6: Tumor necrosis factor-inducible gene 6 protein
